# Tetrafunctional Epoxy Resin-Based Buoyancy Materials: Curing Kinetics and Properties

**DOI:** 10.3390/polym12081732

**Published:** 2020-08-03

**Authors:** Sizhu Yu, Xiaodong Li, Meishuai Zou, Zhiren Li, Shuo Wang, Danhui Wang

**Affiliations:** School of Materials Science and Engineering, Beijing Institute of Technology, Beijing 100081, China; yvsizhu@163.com (S.Y.); bitlxd@bit.edu.cn (X.L.); lzr_bit@163.com (Z.L.); wangshuo@bit.edu.cn (S.W.); wangdh7593@126.com (D.W.)

**Keywords:** epoxy resin, curing kinetics, non-isothermal method, buoyancy material

## Abstract

In order to synthesize a new kind of buoyancy material with high-strength, low-density and low-water-absorption and to study the curing reaction of tetraglycidylamine epoxy resin with an aromatic amine curing agent, the non-isothermal differential scanning calorimeter (DSC) method is used to calculate the curing kinetics parameters of N,N,N′,N′-tetraepoxypropyl-4,4′-diaminodiphenylmethane epoxy resin (AG-80) and the m-xylylenediamine (m-XDA) curing process. Further, buoyancy materials with different volume fractions of hollow glass microsphere (HGM) compounded with a AG-80 epoxy resin matrix were prepared and characterized. The curing kinetics calculation results show that, for the curing reaction of the AG-80/m-XDA system, the apparent activation energy increases with the conversion rates increasing and the reaction model is the Jander equation (three-dimensional diffusion, 3D, n = 1/2). The experimental results show that the density, compressive strength, saturated water absorption and water absorption rate of the composite with 55 v % HGM are 0.668 g·cm^−3^, 107.07 MPa, 0.17% and 0.025 h^−1/2^, respectively. This kind of composite can probably be used as a deep-sea buoyancy material.

## 1. Introduction

In recent years, the resources in land and shallow water have not been able to meet the needs of production and life. These are extremely rich resources, including oil, gas, and minerals, which are stored in deep-sea. Therefore, deep-sea resource exploitation has become a global trend [1,2,3] that cannot function without the support of equipment such as deep submersibles. At present, submersibles generally adopt unpowered floating technology, so buoyancy materials with high-strength and low-density are required to provide part of the net buoyancy [4,5]. The float and sink of submersibles are controlled by the unload and load of buoyancy materials. Considering the working environment of buoyancy materials, high pressure resistance, low density, and low water absorption [6,7,8] are obtained. Buoyancy material is lightweight porous composite material that is prepared by hollow glass microspheres (HGM) as filler that is physically mixed in a polymeric resin matrix [9,10]. Epoxy resin is a common matrix for buoyancy materials because of its excellent comprehensive properties and compatibility [11,12,13,14,15], which exceeds the other thermosetting resin, such as phenolic and polyurethane resins [16,17].

The network of epoxy resin determines the strength, density, saturated water absorption and other properties of buoyancy materials to some extent [18,19,20]. To achieve combined properties, uncured epoxy resin must be converted into a crosslinked structure with curing agents under optimal conditions. Therefore, the crosslink network of epoxy resin is affected by curing reactions once the epoxy oligomer and curing agent are fixed. For the simulation of the curing process, thermal analysis kinetics (TAK) is a common method [21,22,23,24] to analyze the curing reaction of epoxy resin [25,26,27,28]. Kinetic parameters like activation energy and a mechanism function calculated by the TAK method can be used to evaluate some properties of epoxy resin, such as stability [29,30], life-time [31,32] and process [33,34]. Moreover, Mustata et al. [35] studied the thermal curing and degradation kinetics of bisphenol A diglycidyl ether (DGEBA) epoxy resin/amidodiacids cured system in order to analyze the effect of the chemical structure of curing agents on the kinetic of crosslinking reactions and thermal properties by the Kissinger and Ozawa method. The results of the thermal kinetic are consistent with the TG/FT-IR/MS analysis. Therefore, the results derived from the TAK method have a certain accuracy.

In this study, the matrix of buoyancy materials consisted of m-xylylenediamine (m-XDA) cured *N*,*N*,*N*’,*N*’-tetraepoxypropyl-4,4′-diaminodiphenylmethane (AG-80) epoxy resin. The Friedman and Šatava-Šesták methods were used to analyze the apparent activation energy (*E*_a_) and the most probable mechanism function of AG-80/m-XDA system, respectively. The density, compressive strength, saturated water absorption, water absorption rate and cross-sectional morphology of composites with different volume fractions (*V*_H_) of hollow glass microspheres (HGM) were tested as a kind of buoyancy material.

## 2. Materials and Methods

### 2.1. Materials

The *N*,*N*,*N*’,*N*’-tetraepoxypropyl-4,4’-diaminodiphenylmethane epoxy resin (AG-80) with an epoxy value of 0.75–0.85 mol·100 g^−1^ was purchased from Shanghai Institute of Synthetic Resins, Shanghai, China. The m-xylylenediamine (m-XDA) curing agent with 99% purity was obtained from Shanghai Macklin Biochemical Co., Ltd., Shanghai, China. The hollow glass microspheres (HGM) of XLD3000 type used in this study were supplied by 3M Co., Ltd., Sao Paulo, MN, USA. The density and strength of XLD3000 type HGM were 0.23 g·cm^−3^ and 20.67 MPa.

### 2.2. Preparation

The epoxy resin was desiccated and deformed in a vacuum at 80 °C and HGM was dried at 80 °C for 2 h before use, respectively. AG-80 epoxy resin and m-XDA at the mass ratio of 100:26 were mixed well at room temperature. Some of the mixture was used for differential scanning calorimeter (DSC) test. Then, different volume fractions (0, 40, 45, 50, 55 v %) of HGM were mixed with AG-80 and m-XDA mixture at a stirring speed of 10 rpm. After all the HGMs were mixed evenly, the mixture was poured into a mold and placed in a vacuum at room temperature for 50 min. The samples were pre-cured at room temperature for 24 h and then cured at 80 °C for 4 h.

### 2.3. Characterization

The non-isothermal kinetic parameters of the AG-80/m-XDA curing system were calculated based on DSC tests at different heating rates. It was performed by a differential scanning calorimeter (DSC, 204F1, NETZSCH, Selb, Germany) with liquid nitrogen refrigeration. The samples enclosed in aluminum crucible for the DSC test was a mixture of AG-80 epoxy resin and m-XDA at a mass of 10–15 mg. The purge and protective gases were *N*_2_. This step was repeated and the heating rates of the DSC tests were 2.5, 5, 10, 15 and 20 °C·min^−1^, respectively. Moreover, the temperature range, purge gas flow rate and protective gas rate conducted on the DSC tests were 30–180 °C, 20 and 60 mL·min^-1^, respectively.

For the comprehensive properties test of samples with different volume fraction HGM, the density was tested according to Archimedes’ principle and the compressive strength was tested by the electronic universal testing machine (5985, INSTRON, Cambridge, MA, USA) for cylindrical samples of Φ10 mm × 25 mm at a speed of 2 mm·min^−1^. Each final value of compressive strength was the average of five measurements repeatedly. The scanning electron microscope (SEM, TM3000, Hitachi, Tokyo, Japan) was used to observe the dispersing and fracture of HGM in the epoxy resin matrix. The saturated water absorption (*c*_s_) and water absorption rates (*K*) were measured with the square pieces of 50 mm × 50 mm × 2 mm, which were dried at 50 °C for 24 h and cooled to room temperature before testing. Then, the samples were weighed in dry and saturated water situations, respectively. The *c*_s_ was defined as the quotient of the difference between the final quality and the initial quality of sample tested. According to the simplified Fric’s law and *K = c*_s_[16*Dt*/(π*d*^2^)]^1/2^, *K* can be calculated by the following equation:

(1) $$c_{t}=Kt^{1/2},$$

where *c*_s_ is the saturated water absorption, *c*_t_ is water weight gain rate, *t* is the testing time, *d* is the sample thickness, *D* is the diffusion coefficient and *K* is the water absorption rate.

## 3. Results and Discussion

### 3.1. Curing Behaviors and Kinetics

**Figure 1:**
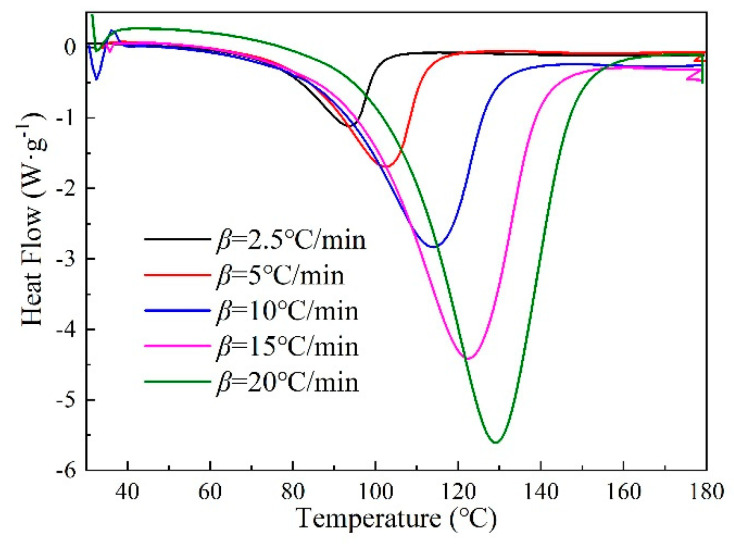
The differential scanning calorimetry (DSC) curves of AG-80/m-xylylenediamine (m-XDA) curing system at different heating rates (*β* = 2.5, 5, 10, 15, and 20 °C·min^−1^) in *N*_2_ atmosphere [37].

The DSC test is a common method for studying curing kinetics [36]. Figure 1 is the DSC curves of AG-80/m-XDA curing system at different heating rates [37]. Most aromatic amine curing agents are solid at room temperature, yet there are more flexible groups in m-XDA molecular, m-XDA is liquid at room temperature. Thus, there is no melting peak in Figure 1. In Figure 1, DSC curves of AG-80/m-XDA curing system at different heating rates all have a single exothermic peak, which indicates that the crosslink reaction occurs between the amine and epoxy groups simultaneously while temperature rises. Additionally, the amine-epoxy addition reaction occurred without side reactions. With an increasing heating rate, the exothermic peaks become sharper and sharper, which indicates that the curing time becomes shortened. Further, characteristic temperatures included initial temperature, peak temperature and final temperature, which are shifted to the high temperature range. This is because at a higher heating rate, the molecular chain cannot move faster as the temperature rises, so the effective collisions cannot occur in time, which is also the reason for the exothermic peak becoming narrow at higher temperatures.

The Friedman and Šatava–Šesták methods were used to study the apparent activation energy (*E*_a_) and reaction mechanism function of the AG-80/m-XDA curing system, respectively. The Friedman method [38] is a type of classic *E*_a_ calculation based on DSC curves, as shown in Equation (2). Through the slope and intercept of the ln(*β*d*α*/d*T*)-1/*T* fitting lines, the values of *E*_a_ corresponding to different conversion degrees (*α*) can be obtained.

(2) $$\ln\left(\frac{\beta d\alpha }{dT}\right)=\ln\left[Af\left(\alpha \right)\right]-\frac{E_{a}}{RT}$$

where *β* is the heating rate, *α* is the conversion degree, *f*(*α*) is the kinetic mechanism function in differential form, *T* is the temperature at different conversion degrees, *A* is the pre-exponential factor, *E*_a_ is the apparent activation energy and R is the universal gas constant.

According to Equation (2), Table 1 shows the values of apparent activation energy at different conversion degrees calculated by the Friedman method. In Table 1, the apparent activation energy of the AG-80/m-XDA curing system decreases with the increase in the conversion degree. The reason is that the reaction mechanisms, in particular the diffusion-controlled reaction kinetics effects, drops the apparent activation energy at high-conversion-degrees. The average value of apparent activation energy at different conversion degrees calculated is 52.20 kJ·mol^−1^, which could be considered to be the apparent activation energy of the whole curing reaction by the Friedman method.

The Šatava–Šesták method [39] is a common method to calculate the most probable mechanism function of the curing system.

(3) $$\mathrm{lg}G\left(\alpha \right)=\mathrm{lg}\frac{AE_{a}}{R\beta }-2.314-0.4567\frac{E_{a}}{RT}$$

where *α* is conversion degree, *G*(*α*) is the integral form of kinetic mechanism function, *A* is the pre-exponential factor, *E*_a_ is the apparent activation energy, R is the universal gas constant, *β* is the heating rates and *T* is the temperature at different conversion degrees.

The 40 kinds of integral forms of the kinetic mechanism function [40], which are commonly used, should be brought into Equation (3). The most probable mechanism function is the value of correlation coefficient value, which is the closest to 1 of lg*G*(*α*)-1/*T* fitting liners [39] through Equation (3) by the Šatava–Šesták method. The DSC curve at the heating rate of 2.5 °C·min^-1^ in Figure 1 was used to calculate the most probable mechanism function by the Šatava–Šesták method. Table 2 listed the higher correlation coefficients reaction model for lg*G*(*α*)-1/*T* fitting liners in 40 forms of the reaction mechanism function by the Šatava–Šesták method.

There are four forms of correlation coefficients in the reaction model that are higher than one by the Šatava–Šesták method. Therefore, combined with the Ozawa method [41], the most probable mechanism function was determined with differences to the *E*_a_ calculated by the two methods. The minimum difference of the four reaction models in Table 2 was the most probable mechanism function [42]. Table 3 shows the value of the apparent activation energy calculated by the Šatava-Šesták method for higher correlation coefficients reaction models in Table 2 and values of apparent activation energy calculated by the Ozawa method, which was 68.09 kJ·mol^−1^. In Table 3, the apparent activation energy of the Jander equation (3D, n = 1/2), calculated by the Šatava–Šesták method, was the closest to the value calculated by the Ozawa method. It was found that Jander equation (3D, n = 1/2) reaction model met all the judging conditions, which was the only mechanism function of AG-80/m-XDA curing reaction. Therefore, the reaction model of the AG/80-m-XDA curing system was the Jander equation, the express of which was *f* (*α*) = 6(1 − *α*) ^2/3^[1 − (1 − *α*) ^1/3^]^1/2^, and the physical meaning underlying the model was three-dimensional diffusion, 3D, *n* = 1/2.

Based on the calculation results of the Friedman and Šatava–Šesták methods, the integral and differential form of the obtained kinetics are as following

(4) $$G\left(\alpha \right)={\left[1-{\left(1-\alpha \right)}^{1/3}\right]}^{1/2},$$

(5) $$f\left(\alpha \right)=6{\left(1-\alpha \right)}^{2/3}{\left[1-{\left(1-\alpha \right)}^{1/3}\right]}^{1/2}.$$

Commonly, the curing kinetics equation is as following:

(6) $$\frac{d\alpha }{dt}=A\exp^{-\frac{E}{RT}}f\left(\alpha \right).$$

Thus, the curing kinetics equation of AG-80/m-XDA is as following:

(7) $$\frac{d\alpha }{dt}=6\times 10^{8.23}\exp^{-\frac{64480}{RT}}{\left(1-\alpha \right)}^{2/3}{\left[1-{\left(1-\alpha \right)}^{1/3}\right]}^{1/2}.$$

### 3.2. Cross-Sectional Morphology Analysis

**Figure 2:**
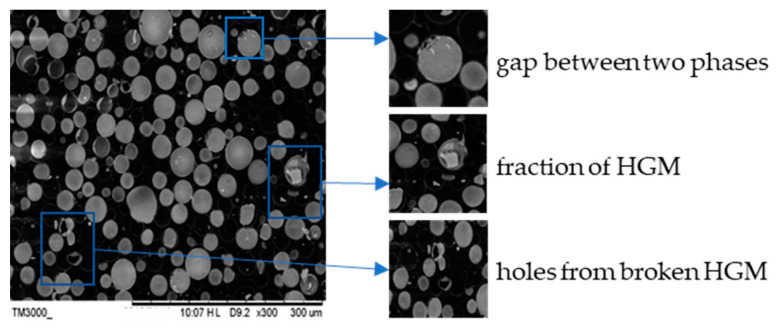
The cross-sectional morphology of AG-80 epoxy resin-based buoyancy material with a 55% volume fraction of hollow glass microspheres (HGM.)

For composites, the lightweight filler of HGM was a kind of inorganic and the cured epoxy resin matrix was a type of organic phase. Therefore, the cohesiveness of the two phases could affect some of the properties of the composite. Further, due to the lower strength of HGM, some of HGM broke during the stirring physical process. The flaw from HGM broken like fragments of HGM and gap between the two phases decreased some of the working properties of the composite. Due to more HGM in composites with *V*_H_ = 55%, the cross-section of the sample with *V*_H_ = 55 v % was observed by SEM. In Figure 2, the HGM was uniformly distributed in the matrix and there was no agglomeration of HGM. The strength of XLD3000 type HGM was about 20.67 MPa, which was far below that of the resin matrix, so some HGM broke during the process. Besides, some of flaws still existed in Figure 2. For the effects of flaws on the working properties of the composites, the fraction of HGM could increase the density and the holes could decrease the density and strength. Further, the space in the gaps and holes could increase the water absorption. The lesser flaw in the composite was that it had no significant agglomeration in the working properties.

### 3.3. Properties of Buoyancy Materials

In the composite equipped in the submersible to provide net buoyancy as floating force, the lower density could supply more buoyancy at the same volume. The density of the resin matrix was higher than that of water, so the HGM at a density of 0.23 g·cm^−3^ as lightweight filler to decrease the density of the composite. Further, the theoretical density of the composite was also calculated. Combined with the SEM test, the deviation of theoretical and tested density could be used to evaluate the process of composites, such as mixing speed, additive amount of HGM for each time and so on. The density calculated (*ρ*_cal_) and tested (*ρ*) at *V*_H_ = 0, 40, 45, 50, and 55 v % is in Table 4. The tested density of the composite with HGM filler was all lower than the density calculated. Even though some of HGM fractions increased the density of the composite, the hole from the stirring process and tiny broken HGM showed more matrix decrease in the density of composites. The deviation between the theoretical and tested results was relatively lower and decreased with HGM content increases. That means that there were few HGM breakages in the preparation process. HGM added in the uncured AG-80/m-XDA system increased the contact area of raw materials [43,44]. For higher curing reaction rates of AG-80 epoxy resin and m-XDA curing agent, the cross-linked network structure was more easily formed, so the viscosity of the mixture became higher. The viscous matrix protected the HGM from breaking. Hence, the deviation became lower with the HGM content increases.

Due to high pressure in deep-sea, the composite must have properties with high strength. The compressive strength (*E*) was tested, as shown in Table 5. The strength of composite decreased with HGM content increases, because the strength of the HGM filler was about 20.67 MPa, which was much lower than the resin matrix. The maximum compressive strength of the composite was determined by the HGM strength and content. For the accumulation of the filler and the stress of the matrix, Turesanyi et al. [45] proposed a semi-empirical formula for the compressive strength of the composites.

(8) $$\sigma_{c}=\sigma_{m}\frac{1-V_{H}}{1+\delta V_{H}}\exp\left(\varepsilon V_{H}\right),$$

where *σ*_c_ is composite compressive strength; *σ*_m_ is the resin matrix strength; *V*_H_ is the HGM volume fraction; *δ* is the filler factor; for HGM, *δ* = 2.5; and *ε* is the interface bonding ability of HGM and matrix. The system had no bonding and was well bonded for *ε* = 0 and *ε* = 3, respectively.

**Figure 3:**
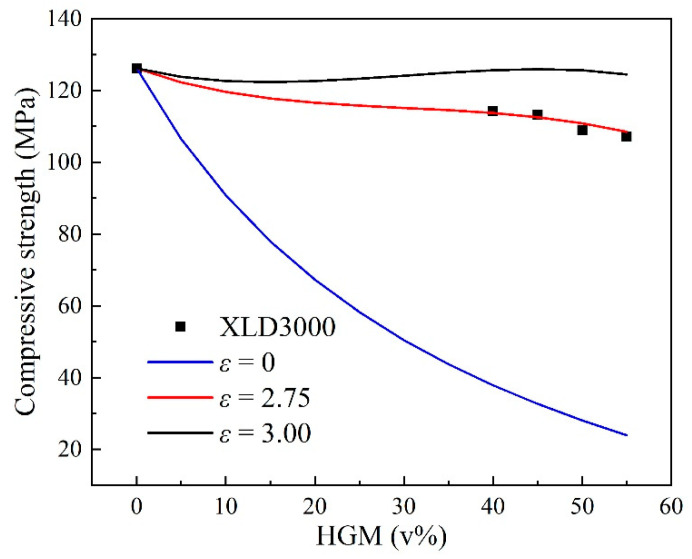
The curves of the compressive strength with the volume fraction for AG-80 epoxy resin-based buoyancy materials.

Figure 3 shows the relationship between compressive strength and HGM volume fraction for AG-80 epoxy resin-based buoyancy materials, according to Equation (8). In Figure 3, the relationship of compressive strength and HGM volume fraction can be described well at the value of *ε* = 2.75, so the adhesion of the matrix and HGM was much better, according to the Turesanyi method, by Equation (8). Besides, no clearly separated bubbles appeared in the cross-sectional morphology of the composite (Figure 2), which is consistent with the results obtained by the fitting curves of the compressive strength and the lower deviation of density values for buoyancy materials. Further, the compressive strength of the composite calculated in accordance with Equation (8) is also shown in Table 5. The tiny deviation in compressive strength tested and calculated was due to the flaw of HGM in being crushed and bubble mixed. As the working properties of buoyancy material was low-density and high-strength, the HGM added could decrease the density of the composite effectively, but the low strength of the HGM also decreased the compressive strength of the composite. The ratio of strength and density (*E*/*ρ*) was defined to evaluate the possibility used as buoyancy materials. In Table 5, the values of *E*/*ρ* increases with the increasing of the HGM volume fraction, which indicates the XLD3000 type of HGM can decrease the density and maintain the mechanical properties of buoyancy materials to some extent.

The buoyancy materials can provide net buoyancy force for submersibles in deep-sea, so it must be low water absorption. The HGM added can increase viscosity of the mixture, so it had no ability to prepare samples with an HGM distribution and no bubbles without being thin at HGM volume fractions higher than 55%. In Table 5, the sample with 55 v % HGM had the highest value of *E*/*ρ*, so the saturated water absorption of which was tested. Through Fric’s law, saturated water absorption (*c*_s_) and water absorption rate (*K*) values of composites with *V*_H_ = 55 v % were 0.17% and 0.025 h^−1/2^, respectively. There were free volumes between the cured epoxy resin molecular segments and chains, in which water molecules could diffuse and penetrate. Further, for the curing reaction of AG-80/m-XDA, the reaction of epoxy group with the aromatic amine curing agent produced a hydroxyl group. The hydrogen bonds could be formed through the hydroxyl group and water molecules, which could have reduced the hydrophobic of the buoyancy materials. As for the filters of buoyancy materials, the water absorption of HGM was deficient because of its inorganic structure. However, the flaws of like holes and gaps between the matrix and HGM shown in Figure 2 can also diminish the hydrophobic properties of composites.

## 4. Conclusions

A new buoyancy material with high-strength and low-density was prepared by the composition of m-XDA cured AG-80 epoxy resin and HGM. For curing kinetics study, the apparent activation energy decreases with the increasing of the conversion degree of AG-80/m-XDA system and the reaction model is the Jander equation. The expression of the Jander equation is *f*(*α*) = 6(1 − *α*) ^2/3^[1 − (1 − *α*) ^1/3^]^1/2^, and the physical meaning is three-dimensional diffusion (3D, *n* = 1/2). Compounded with different volume fractions (*V*_H_ = 0, 40, 45, 50, and 55 v %) of HGM, the density and compressive strength of composites decrease with the increasing of the HGM volume fraction. However, the value of the strength density ratio (*E*/*ρ*) increases with the increasing of the HGM volume fraction. Due to the viscosity of the mixture becoming higher, the maximum amount of HGM added to the AG-80 resin matrix was *V*_H_ = 55 v % without diluent. For the composite with 55 v % HGM, the density, uniaxial compressive strength, saturated water absorption and water absorption rate were 0.668 g·cm^−3^, 107.07 MPa, 0.17% and 0.025 h^−1/2^, respectively. The composite material with AG-80 epoxy resin as the matrix and HGM as the light filler had the properties of high strength, low density and low water absorption in this study, so it had the possibility of being used as a deep-sea buoyancy material.

## Figures and Tables

**Table 1:** The apparent activation energy values at different conversion degrees obtained by the Friedman method.

*α*	0.1	0.2	0.3	0.4	0.5	0.6	0.7	0.8	0.9
*E*_a_/kJ·mol^−1^	60.37	58.14	57.22	55.71	54.01	51.00	47.41	42.56	43.35

**Table 2:** The highest correlation coefficients reaction model for lg*G*(*α*)-1/*T* fitting liners in 40 forms of the reaction mechanism function obtained by the Šatava–Šesták method.

Function Name	Mechanism	*G*(*α*)	*f*(*α*)	*r* ^2^
Jander equation	3D, *n* = 1/2	[1 − (1 − α)^1/3^]^1/2^	6(1 − α)^2/3^[1-(1 − α)^1/3^]^1/2^	0.9999
Jander equation	3D, D_3_, *n* = 2	[1 − (1 − α)^1/3^]^2^	3/2(1 − α)^2/3^[1 − (1 − α)^1/3^]^−1^	0.9999
Order of reaction	*n* = 1/4	1 − (1 − α)^1/4^	4(1 − α)^3/4^	0.9999
Shrinkage spherical (volume)	R_3_, *n* = 1/3	1 − (1 − α)^1/3^	3(1 − α)^2/3^	0.9999

**Table 3:** The kinetic parameters calculated by the Šatava–Šesták method for four kinds of higher correlation coefficients reaction models by lg*G*(*α*)-1/T fitting liners.

*G*(*α*)	*E*_a_ (kJ·mol^−1^)	lg*A*	*r* ^2^
[1 − (1 − α)^1/3^]^1/2^	64.48	8.23	0.9999
[1 − (1 − α)^1/3^]^2^	257.91	35.86	0.9999
1 − (1 − α)^1/4^	128.96	17.34	0.9999
1 − (1 − α)^1/3^	128.96	17.82	0.9999

**Table 4:** The theoretical and tested density of the composite at *V*_H_ = 0, 40, 45, 50, and 55 v%.

*V*_H_ (v%)	0	40	45	50	55
*ρ* (g·cm^−3^)	1.208	0.802	0.746	0.711	0.668
*ρ*_cal_ (g·cm^−3^)	——	0.817	0.768	0.719	0.670

**Table 5:** The mechanical properties of composites with different volume fraction HGMs.

*V*_H_/%	0	40	45	50	55
*E*/MPa	126.14	114.20	113.27	108.99	107.07
*E*_cal_/MPa	——	113.68	112.53	110.87	108.46
*E*/*ρ*	104.42	142.29	151.84	153.29	160.28
